# Synergistic Effect of Vaginal Trauma and Ovariectomy in a Murine Model of Stress Urinary Incontinence: Upregulation of Urethral Nitric Oxide Synthases and Estrogen Receptors

**DOI:** 10.1155/2014/314846

**Published:** 2014-08-31

**Authors:** Huey-Yi Chen, Wen-Chi Chen, Yu-Ning Lin, Yung-Hsiang Chen

**Affiliations:** ^1^Graduate Institute of Integrated Medicine, College of Chinese Medicine, Research Center for Chinese Medicine & Acupuncture, China Medical University, Taichung 40402, Taiwan; ^2^Sex Hormone Research Center, Departments of Obstetrics and Gynecology, Urology, and Medical Research, China Medical University Hospital, Taichung 40447, Taiwan

## Abstract

The molecular mechanisms underlying stress urinary incontinence (SUI) are unclear. We aimed to evaluate the molecular alterations in mice urethras following vaginal trauma and ovariectomy (OVX). Twenty-four virgin female mice were equally distributed into four groups: noninstrumented control; vaginal distension (VD) group; OVX group; and VD + OVX group. Changes in leak point pressures (LPPs), genital tract morphology, body weight gain, plasma 17*β*-estradiol level and expressions of neuronal nitric oxide synthase (nNOS), induced nitric oxide synthase (iNOS), and estrogen receptors (ERs—ERα and ER*β*) were analyzed. Three weeks after VD, the four groups differed significantly in genital size and body weight gain. Compared with the control group, the plasma estradiol levels were significantly decreased in the OVX and VD + OVX groups, and LPPs were significantly decreased in all three groups. nNOS, iNOS, and ERα expressions in the urethra were significantly increased in the VD and VD + OVX groups, whereas ER*β* expression was significantly increased only in the VD + OVX group. These results show that SUI following vaginal trauma and OVX involves urethral upregulations of nNOS, iNOS, and ERs, suggesting that NO- and ER-mediated signaling might play a role in the synergistic effect of birth trauma and OVX-related SUI pathogenesis.

## 1. Introduction

Modulation in the contractile response of smooth muscle underlies important pathological conditions such as incontinence and hypertension. These disorders are also frequently encountered in the aged population [1]. Inflammation and oxidative stress are key features in the clinical manifestations of smooth muscle-related disorders [1, 2], including stress urinary incontinence (SUI). SUI is a common urological disease defined as the involuntary leakage of urine under stress conditions such as coughing and sneezing [3]. The effects of birth trauma [4], menopause, and aging may contribute to the development of SUI [5].

Although the treatment of SUI has improved [6], its underlying molecular mechanisms remain unclear. Studies on the effect of birth trauma and menopause on the continence mechanism are lacking because of the restricted availability of human tissue. In this study, we used virgin female mice [7, 8] to analyze the effects of vaginal distension (VD; simulated birth trauma) [9, 10] and hormone deficiency (these two factors known to be important in SUI) on the vagina and urethra. VD simulates the effects of birth trauma [9] and ovariectomy (OVX) simulates the hormone deficiency that occurs after menopause [7].

Birth trauma from vaginal delivery may cause ischemic damage to the urogenital tract [11]. Ischemia induces nitric oxide synthase (NOS) expression; this increases NO synthesis, resulting in urethral relaxation [12–14]. Estrogen actions are mediated by estrogen receptors (ERs) [15, 16], which are encoded by two distinct genes—ERα and ER*β*. Although treatment with 17*β*-estradiol results in increasing urethral tone through the local inhibition of NOS expression, the mechanism by which urethral tone is increased by estradiol through the estrogen receptor subtypes, that is, ERα and ER*β*, is unclear.

Our general goal was to understand the molecular mechanisms related to SUI following simulated birth trauma and OVX. On the basis of the aforementioned findings [17], we hypothesized the following: (1) simulated birth trauma and OVX decrease leak point pressures (LPPs) and plasma estradiol levels; (2) simulated birth trauma and OVX induce atrophy of the urogenital tract and the expression of neuronal nitric oxide synthase (nNOS) with induced nitric oxide synthase (iNOS); (3) ERα and ER*β* expression are altered by simulated birth trauma and OVX in a mouse model of SUI. To test these hypotheses, we designed the present study with the following aims: (1) to analyze LPPs, morphology of the urogenital tract, and plasma estradiol levels in C57BL/6 mice after VD and/or OVX; (2) to identify the induction of nNOS and iNOS expression by simulated birth trauma and/or OVX using immunofluorescence staining and Western blot analysis; and (3) to characterize alterations in ERα and ER*β* expression by simulated birth trauma and/or OVX using immunofluorescence staining and Western blot analysis.

## 2. Materials and Methods

### 2.1. Animals and Experimental Design

Twenty-four virgin female mice (aged 6–8 weeks, weight 25–40 g) were randomly assigned to 4 groups: (1) noninstrumented control; (2) VD (8 mm dilator, compatible with the diameter of a new-born mouse head); (3) OVX group; and (4) VD + OVX group. Sham operations or OVX was performed on the mice in these 4 groups, 2 days after VD (Day 2). Mice underwent suprapubic bladder tubing (SPT) placement 17 days after the surgery (Day 19). LPPs were assessed in these mice under urethane [1 g/kg, intraperitoneal (i.p.)] anaesthesia 2 days after SPT (Day 21). The noninstrumented control group did not undergo VD but did undergo SPT placement and LPP measurement. The animals were sacrificed after examining LPPs, morphology of the urogenital tract, and plasma estradiol levels, and the urethras were removed for immunofluorescence staining and Western blot analysis. All experimental protocols were approved by the Institutional Animal Care and Use Committee of China Medical University.

### 2.2. Vaginal Distension

Mice in the 8 mm VD groups were anesthetized with 1.5% isoflurane. To avoid rupturing the vagina, vaginal accommodation of Hegar's dilators was achieved by sequentially inserting and removing Hegar's dilators of increasing size that were lubricated with Surgilube (Fougera, Melville, NY). Subsequently, an 8-mm dilator was lubricated and inserted into the vagina [18–20]. After 1 h, the 8-mm dilator was removed and the animal was allowed to awaken from the anaesthesia spontaneously. The noninstrumented control group did not undergo vaginal dilation.

### 2.3. Ovariectomy or Sham Operation

Mice undergoing OVX or sham operation were anesthetized with 1.5% isoflurane. In groups 1 and 2, a midline longitudinal abdominal incision was made and closed with 2-0 silk sutures. In groups 3 and 4, both ovaries were excised through a midline longitudinal abdominal incision that was closed with 2-0 silk sutures. The ovaries were rinsed in phosphate-buffered saline (PBS), fixed in 4% formaldehyde/PBS overnight, and processed for embedding in paraffin blocks, according to histological methodology. Cross sections which were perpendicular to the largest axis of the structures to be analysed were cut at 4-*μ*m thickness. Sections were stained with hematoxylin and eosin (H&E) [21, 22]. A light microscope with a 10x ocular and a 40x objective lens was used to analyse the slices.

### 2.4. Suprapubic Tube Implantation

The surgical procedure was carried out under isoflurane anaesthesia according to the methods previously described [18–20, 23]. An SPT (PE-10 tubing, Clay Adams, Parsippany, NJ, USA) was implanted in the bladder 19 days after VD (Day 19). The key points of the operation were as follows: (1) a midline longitudinal abdominal incision was made 0.5 cm above the urethral meatus; (2) a small incision was made in the bladder wall, and PE-10 tubing with a flared tip was implanted in the bladder dome; and (3) the purse-string suture of 8-0 silk was tightened around the catheter which was tunnelled subcutaneously to the neck, where it exited the skin.

### 2.5. Leak Point Pressure Measurement

LPPs were assessed in these mice under urethane anaesthesia, 2 days after implanting the bladder catheter (3 weeks after VD, Day 21). The bladder catheter was connected to both a syringe pump and a pressure transducer. Pressure and force transducer signals were amplified and digitized for computer data collection at 10 samples per second (PowerLab, ADInstruments, Bella Vista, Australia). The mice were placed in a supine position at the level of 0 pressure while their bladders were filled with room temperature saline at 1 mL/h through the bladder catheter. The bladder was emptied manually using Credé's maneuver if a mouse voided. The average bladder capacity of each mouse was determined after 3–5 voiding cycles, and the LPPs were measured as previously described [18–20, 23]. Briefly, gentle pressure was applied with 1 finger to the mouse's abdomen when half-bladder capacity was reached. Pressure was gently increased until urine leaked, at which time the externally applied pressure was immediately removed. The peak bladder pressure was taken as the LPP. At least 3 LPPs were obtained for each animal, and the mean LPP was calculated [24, 25].

### 2.6. Genital Size, Body Weight, and Plasma Estradiol

The morphology and size of genital tract were examined (genital tract length (cm) and weight (mg)). The body weight before VD (Day 0) and 3 weeks after VD (Day 21) was assessed. Serum samples were taken from all animals before death (Day 21). Plasma estradiol was extracted first under diethyl ether and was measured with a commercially available ELISA kit.

### 2.7. Immunofluorescence Staining

The mice in all 4 groups were sacrificed immediately after completing the LPP measurements, and the urethras were harvested. The middle one-third portions of the urethras were examined with immunofluorescence staining. Each tissue sample was embedded in Tissue-Tek Optimal Cutting Temperature (OCT) Compound (Torrance, CA, USA) and then frozen, and 8-*μ*m cryostat sections were cut and used for immunofluorescence staining of nNOS, iNOS, ERα, and ER*β*. For immunofluorescence staining, the sections were permeabilized with 0.05% Triton X-100 for 5 min and blocked with 5% normal bovine serum albumin in PBS for 1 h at room temperature [26, 27]. The sections were incubated with a primary antibody (goat polyclonal to nNOS, 1 : 300 dilution, Abcam (ab1376), Cambridge, UK; rabbit polyclonal to iNOS, 1 : 50 dilution, Abcam (ab3523); mouse monoclonal to ERα, 1 : 300 dilution, Abcam (ab2746); or rabbit polyclonal to ER*β*, 1 : 180 dilution, Abcam (ab3577)) overnight at 4°C. The sections were washed 3 times with PBS, incubated with fluorescein isothiocyanate-conjugated secondary antibody (donkey anti-goat IgG conjugate, 1 : 200 dilution, Invitrogen (A11055), USA; goat anti-rabbit IgG conjugate, 1 : 150 dilution, ZYMED (81-6111); or goat anti-mouse IgG conjugate, 1 : 300 dilution, ZYMED (81-6511)) for 1 h at room temperature, and viewed under fluorescence microscopy.

### 2.8. Western Blot Analysis

Urethral protein samples were prepared by homogenization of cells in a tissue extraction reagent (Invitrogen, USA). Cell lysates containing 100 *μ*g of protein were subjected to 10% sodium dodecyl sulfate polyacrylamide gel electrophoresis and were transferred to a polyvinylidene fluoride membrane (Millipore Corp, Bedford, MA, USA). The membrane was stained with Ponceau S to verify the integrity of the transferred proteins and to monitor the unbiased transfer of all protein samples. Detection of nNOS, iNOS, ERα, ER*β*, and glyceraldehyde 3-phosphate dehydrogenase (GAPDH) on the membranes was performed with an electrochemiluminescence kit (Amersham Life Sciences Inc., Arlington Heights, IL, USA) [28–30] using the following antibodies: goat polyclonal to nNOS, 1 : 300 dilution, Abcam (ab1376); rabbit polyclonal to iNOS, 1 : 200 dilution, Abcam (ab3523); mouse monoclonal to ERα, 1 : 1000 dilution, Abcam (ab2746); or rabbit polyclonal to ER*β*, 1 : 500 dilution, Abcam (ab3577). The intensity of each band was quantified using a densitometer (Molecular Dynamics, Sunnyvale, CA).

### 2.9. Statistical Analyses

Data are presented as mean ± standard error of mean (SEM) for each group. Statistical differences among groups were determined by one-way analysis of variance (ANOVA) followed by Fisher's least significant difference (LSD) as a posthoc test. All statistical tests were 2-sided. A *P* value of less than 0.05 was considered statistically significant. All calculations were performed using the Statistical Package for the Social Sciences (SPSS for Windows, SPSS Inc., Chicago, IL, USA).

## 3. Results

### 3.1. Genital Tract Length, Genital Weight, and Body Weight Gain

**Figure 1:**
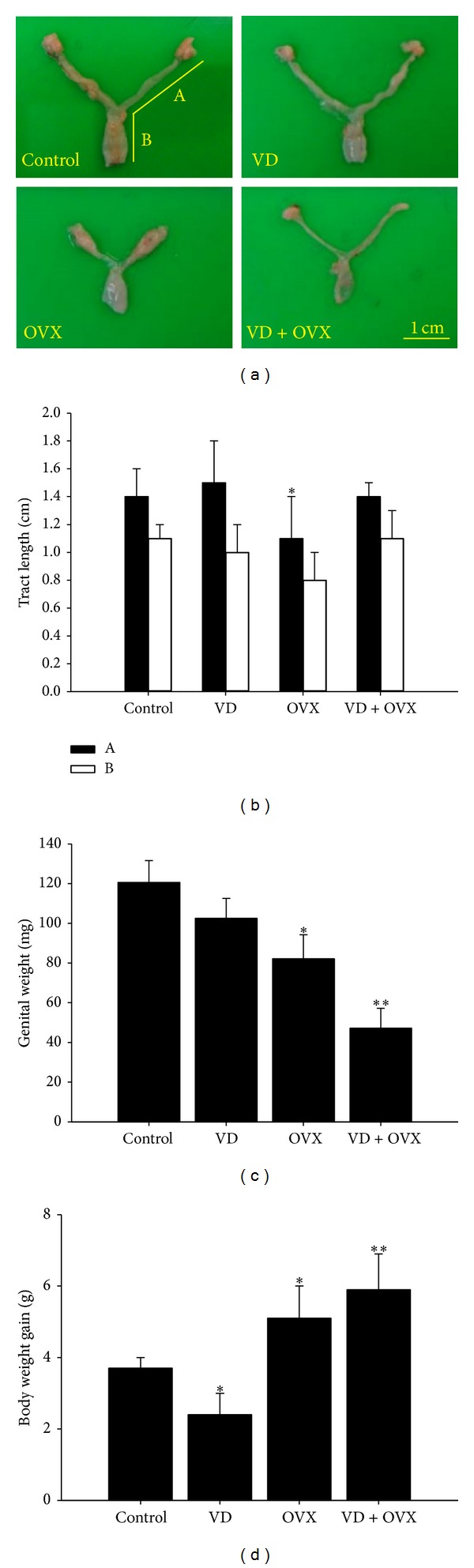
(a) Morphology of the genital tract in the different groups (A and B reflect genital tract lengths in (b)). The four groups differed significantly in (b) genital tract length, (c) genital weight, and (d) body weight gain. Data are expressed as mean ± SEM; *P < 0.05 compared with control. **P < 0.01 compared with control.

The four groups differed significantly in genital tract length, genital weight, and body weight gain. Genital size was significantly decreased in the OVX (*P* < 0.05) and VD + OVX (*P* < 0.01) groups (Figures 1(a)–1(c)). Body weight gain was significantly increased in the OVX (*P* < 0.05) and VD + OVX (*P* < 0.01) groups but decreased in the VD group (*P* < 0.05) (Figure 1(d)).

### 3.2. Plasma Estradiol Level

**Figure 2:**
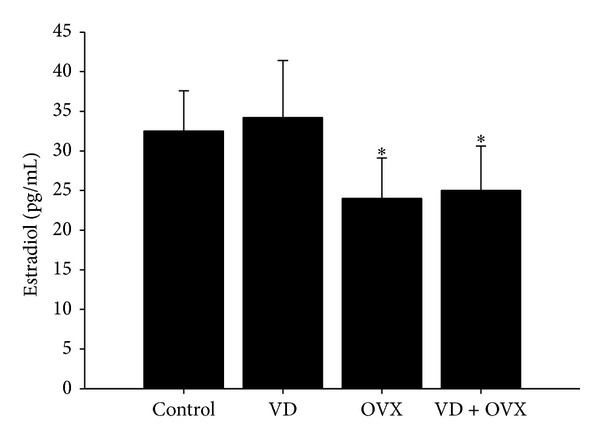
Plasmas estradiol in the different groups. Each bar represents the mean ± SEM of six individual mice. *P < 0.05 compared to control group.

The plasma estradiol level on Day 21 was significantly decreased in the OVX and VD + OVX groups (*P* < 0.05) as compared with that in control group (Figure 2).

### 3.3. LPP Values

**Figure 3:**
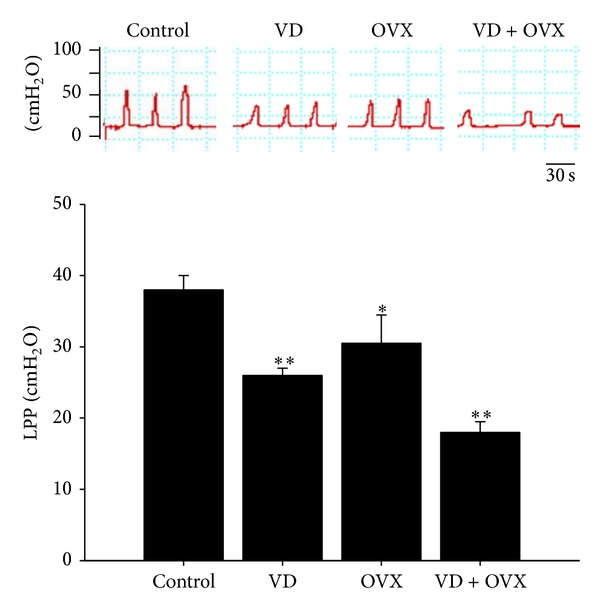
LPP values in the different groups. Each bar represents the mean ± SEM of six individual mice. *P < 0.05 compared to control group; **P < 0.01 compared to control group.

LPP values on Day 21 were significantly decreased in the VD (*P* < 0.01), OVX (*P* < 0.05), and VD + OVX (*P* < 0.01) groups as compared with those in the control group (Figure 3).

### 3.4. Expression of nNOS, iNOS, ERα, and ER*β*

**Figure 4:**
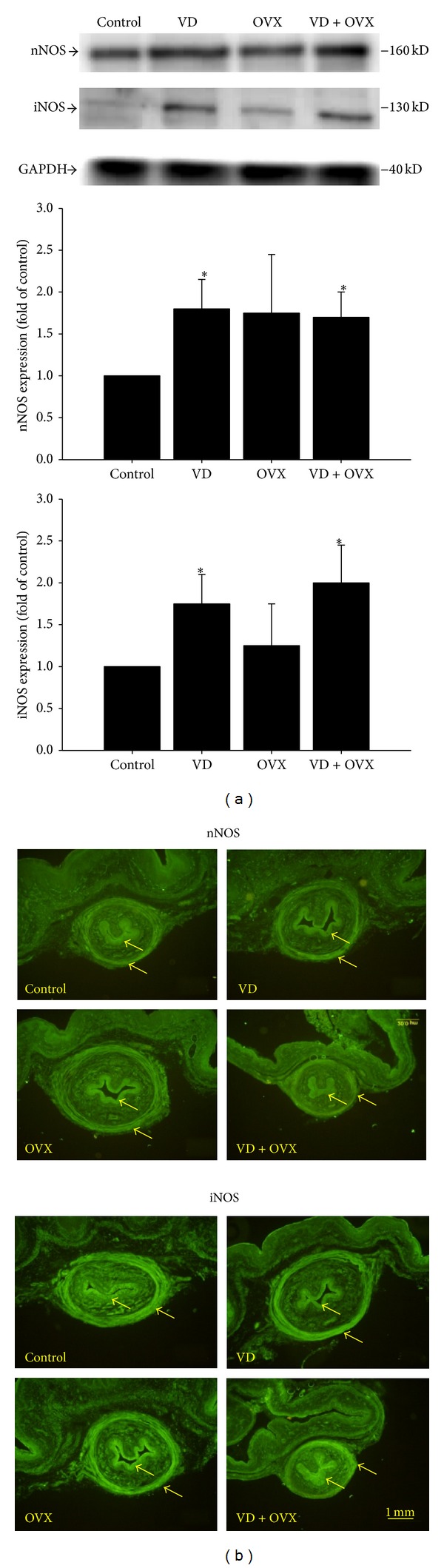
nNOS and iNOS expressions in the transverse sections of the midurethra as indicated by (a) Western blot and (b) immunofluorescence staining in the different groups. The values are calculated by fold of control and expressed as mean ± SEM of six individual mice. *P < 0.05 compared to control group.

**Figure 5:**
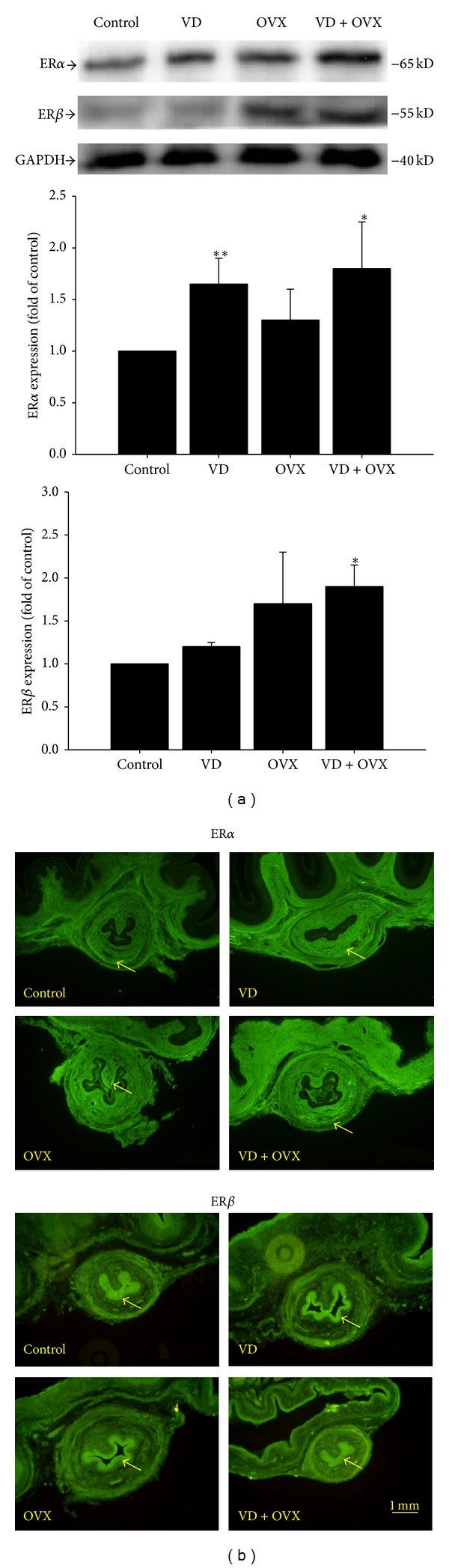
ERα and ER*β* expressions in the transverse sections of the midurethra as indicated by (a) Western blot and (b) immunofluorescence staining in the different groups. The values are calculated by fold of control and expressed as mean ± SEM of six individual mice. *P < 0.05 compared to control group; **P < 0.01 compared to control group.

The expression levels and locations of nNOS, iNOS, ERα, and ER*β* are shown in Figures 4 and 5, respectively. Both nNOS and iNOS expressions were significantly increased in the VD (*P* < 0.05) and VD + OVX (*P* < 0.05) groups as compared with those in the control group (Figures 4(a) and 4(b)). ERα expression was significantly increased in the VD (*P* < 0.01) and VD + OVX (*P* < 0.05) groups, whereas ER*β* expression was significantly increased only in the VD + OVX (*P* < 0.05) group (Figures 5(a) and 5(b)).

## 4. Discussion

Birth trauma occurs as a direct result of a higher ratio of the baby's head to the birth canal in humans. The pressure on the vaginal sidewall can reach 240 cmH_2_O during the peak contractions if active labor is maintained for more than 30 min, which can cause microcirculatory ischemia and overstretching of the pelvic floor muscles, pubourethral ligaments, and nerve tissue; these combined events can lead to SUI [31–33]. Birth trauma and menopause play important roles in the development of SUI [34]. In light of the paucity of human urethral tissue available for analysis, our animal models of VD- and OVX-induced SUI represent a reasonable proxy for the study of the urethral effects of labor and menopause.

In the present study, LPPs were significantly decreased in the VD, OVX, and VD + OVX groups, indicating that vaginal trauma and OVX cause injury to the urethra. These findings agree with those of a previous report [7]. VD and OVX, which induce ischemia, stretch injury, and estrogen deficiency, may cause alterations in the composition and function of the lower urinary tract (LUT) tissues [35].

NOS expression is activated by ischemia [12, 13], and NOS has also been found in the urethra and NO has been implicated as one of the neurotransmitters involved in urethral relaxation [36]. The present study showed that nNOS/iNOS expression in the urethra was significantly increased in the VD and OVX groups as compared with those in the control group. These findings suggest that NOS can be activated by vaginal trauma and OVX. The overexpression of iNOS reportedly increases NO synthesis, resulting in urethral relaxation [12, 13]. Estrogen deficiency increases NOS expression and decreases urethral trophicity [37]. Sievert et al. had found a high number of nNOS-positive nerves in the smooth muscle bundles of the urethra and bladder neck of the experimental rats [38]. The significance of their finding is unclear because the role of NO in urethral relaxation remains controversial [39, 40]. Nevertheless, we propose that OVX after vaginal trauma may synergistically induce SUI, which is related to NOS upregulation.

Furthermore, estrogen deficiency causes hot flushes, sleep disturbances, urogenital atrophy, weight gain, osteoporosis, and increased rates of myocardial infarction [41]. Estrogen is known to have an important role in the function of the LUT and estrogen receptors have been demonstrated in the vagina, urethra, bladder, and pelvic floor musculature. In addition estrogen deficiency occurring following the menopause is known to cause atrophic change and may be associated with LUT symptoms. These may also coexist with symptoms of urogenital atrophy. Epidemiological studies have implicated estrogen deficiency in the etiology of LUT symptoms with 70% of women relating the onset of urinary incontinence to their final menstrual period [42]. Our findings reveal that genital atrophy and weight gain occurred in the OVX and VD + OVX groups, thus suggesting that OVX can induce estrogen deficiency. ER expression levels in the urethra were significantly increased in the VD + OVX group. Estrogen regulates ER expression [43] and augments stress-induced signaling and gene expression [41]. Thus, these results suggest the expression of ERs is activated by OVX after vaginal trauma which induces stress.

Our study has certain limitations. First, the pelvic floor structure of the mouse—quadruped with a lax abdominal wall—differs from that of a human female. Therefore, the results should be extrapolated to human subjects with caution. Second, the urodynamic studies were conducted under anesthesia. Fortunately, no subjects in the present study had bladder instability, implying no detrusor overactivity; this gives credence to our interpretation of fluid expulsion in the absence of increased bladder pressure as evidence of SUI. Third, we only observed changes in the expressions of nNOS, iNOS, and ERs. The use of gene chip and proteomic analyses would facilitate a comprehensive assessment of genome expression in the given tissues and would possibly reveal other mechanisms of VD + OVX-induced incontinence; therefore, future studies should employ these methods. Fourth, most studies concerning the effects of birth trauma on the animal pelvic floor have used pregnant rats [44]. Compared to nongravid rats, pregnancy confers a different hormonal status with other accompanying changes [45]. As the pelvic structures affected by pregnancy hormones may respond differently to trauma, this study reflects vaginal trauma, not birth trauma.

## 5. Conclusions

The results of the present study suggest that SUI following vaginal trauma and OVX involves urethral upregulation of nNOS/iNOS and ERs. Upregulation of NOS might play a role in the synergistic effect of SUI pathogenesis following simulated birth trauma and OVX. This information could offer interesting clues regarding the pathogenesis of SUI and suggest several avenues for novel research and potential new therapies.
